# An Insight into Dowry Deaths: The Untold Stigma and Torment of a Social Evil

**DOI:** 10.7759/cureus.76348

**Published:** 2024-12-24

**Authors:** Sanjeet Kumar, Kumar Shubhendu, Sanjay Kumar, Anand Kumar

**Affiliations:** 1 Department of Forensic Medicine and Toxicology, Shahid Nirmal Mahto Medical College, Dhanbad, IND; 2 Department of Forensic Medicine and Toxicology, Rajendra Institute of Medical Sciences, Ranchi, IND

**Keywords:** dowry, marriage, postmortem, socio demographic factors, women of childbearing age

## Abstract

Background and objective

The institution of marriage is an essential building block of societal structure, acting as a catalyst for joyous celebrations and fresh beginnings. Nonetheless, a persistent problem related to marriage, especially from the viewpoint of women in Indian society, is the dowry system. Despite extensive criticism and opposition, the custom remains prevalent, manifesting in subtle as well as in overt ways. We still see a heightened and intensified manifestation of the evils of this system due to the problematic approach of the general public. The majority of the population still considers the practice of demanding and expecting bride prices to be acceptable and even justified. This study aimed to grasp the interconnected sociodemographic factors and suggest potential measures for intervention.

Material and methods

The study was undertaken from April 2021 to March 2022 at the Department of Forensic Medicine and Toxicology, Rajendra Institute of Medical Sciences, Ranchi, Jharkhand. A total of 3840 postmortem examinations were conducted during this period; among these, 225 cases pertained to women of childbearing age, with 12 instances meeting the specific criteria established for alleged dowry-related fatalities.

Results

A considerable proportion of women among suspected dowry-related fatalities were in the age range of 16-26 years, engaged in domestic duties, adhered to the Hindu faith, belonged to the middle socioeconomic class, lacked professional qualifications, and belonged to either joint or nuclear family setups, and hailed from both rural or urban environments. The marriages, predominantly arranged, had typically lasted for less than two years. The bulk of dowry-linked fatalities exhibited characteristics indicative of homicide, with burn injuries standing out as the most common cause of death. A noteworthy number of these incidents occurred either at the in-laws' residence or the husband's abode, with the in-laws and the husband themselves often identified as the primary culprits in the majority of cases. In such scenarios, they were either directly responsible for the demise or compelled the victim to end her own life, usually due to demands for dowry and ill-treatment.

Conclusions

A multitude of factors are markedly associated with the rising occurrences of dowry-related fatalities, including the level of education attained by women, the prevalence of extended family structures, the dynamics within the matrimonial relationship, the employment status of the spouse, women's dependency on their husbands and/or in-laws, socioeconomic conditions, and cases of marital unfaithfulness. More stringent enactment of the existing laws against offenders, the enhancement of educational and professional opportunities for women, and the improvement of their overall socioeconomic status are essential measures for addressing this societal affliction.

## Introduction

Dowry-related deaths, a critical societal concern, have been extensively examined in the field of forensic medicine, to comprehend their epidemiology, causes, and medico-legal implications. These fatalities, frequently affecting young married females (without neglecting the fact that child marriages are also seen in the Indian scenario), are predominantly attributed to the demands for dowry in various forms, a custom deeply entrenched in certain societies. A notable proportion of these fatalities are considered to be homicides, utilizing means such as burning, poisoning, and strangulation, often camouflaged as suicides or mishaps to avoid legal repercussions [1-5]. Studies suggest that the frequency of dowry-related fatalities fluctuates in accordance with the level of economic advancement in various geographical areas. In regions with limited economic progress, customary informal structures or institutions dictate conduct, leading to high levels of dowry-related fatalities. Conversely, with economic and social development, the occurrence of dowry-related fatalities generally diminishes, indicating a multifaceted interplay between economic standing and societal conventions [6].

Epidemiological studies in India have demonstrated that the majority of individuals affected by this tragedy belong to the youthful demographic, are adherents of Hinduism, and married women hailing from low to lower-middle-class backgrounds, particularly within the age range of 18-32 years. Typically, the principal perpetrators are identified to be the spouse and his relatives, with the incidents predominantly taking place within the domestic confines of the victim. The use of fire, notably with kerosene as an accelerant, is frequently reported, leading to significant burn traumas and deaths. Furthermore, forensic analysis plays a pivotal role in distinguishing between unintentional, self-inflicted, and intentional fatalities, thereby facilitating the legal proceedings [7,8].

The National Crime Records Bureau (NCRB) has documented that there were 6450 instances of dowry-related deaths in India in 2022, indicating a slight decline from the figures of 6753 and 6966 in 2021 and 2020, respectively. The rate of criminal activities related to dowry deaths has remained constant at one per 100,000 women over the past two years, slightly higher than the rate of 1.1 per 100,000 women in 2020. In the state of Jharkhand, there were 208 reported cases in 2022, 281 in 2021, and 275 in 2020, with a corresponding crime rate of 1.1 per 100,000 in 2022 and 1.5 in both 2021 and 2020. The cases filed under the Dowry Prohibition Act of 1961 in India have exhibited an upward trajectory in recent times, standing at 13,479 and 13,568 in 2022 and 2021, respectively, in contrast to the figure of 10,366 in 2020, closely resembling the patterns observed in Jharkhand [9-11].

The response of the legal system to dowry-related fatalities entails the utilization of specific provisions within the Indian Penal Code (IPC)/Bharatiya Nyaya Sanhita (BNS), to deter such offenses. Nevertheless, the high prevalence of dowry-related deaths underscores the need for more rigorous efforts to effectively implement the relevant laws along with actions aimed at safeguarding women. Recommendations have been made for the integration of digital technologies and artificial intelligence (AI) methodologies within the legal framework to hasten the dispensation of justice and address the accumulation of pending cases [12], especially those related to dowry deaths. To summarize, dowry-related fatalities constitute a notable societal concern, underscoring the necessity for a comprehensive approach that encompasses legal revisions, socioeconomic advancement, and educational initiatives to alleviate this problem.

## Materials and methods

This cross-sectional study was conducted between April 2021 and March 2022 in the Department of Forensic Medicine and Toxicology at Rajendra Institute of Medical Sciences, a tertiary healthcare facility situated in Ranchi, the capital city of the state of Jharkhand in India. During the study period, a total of 3840 medicolegal autopsies were conducted; 225 of these pertained to females within the reproductive age bracket, i.e., 15-44 years, out of which 12 cases were found to align with the predetermined criteria for dowry-related fatalities that were registered under section 304 B IPC (section 80 BNS), who had died within seven years of marriage. Highly decomposed bodies and cases without proper identification documentation were excluded.

The detailed data related to age, religious affiliation, familial structure, place of residency, socioeconomic standing, professional field, level of educational attainment, type of marriage, dowry demands before marriage, duration of marital union, children, as well as the circumstances and factors leading to the occurrence of death, viz. provocative factors, time and place of incidence, type of offenders/instigators, place of death, along with cause and manner of death were obtained utilizing a pre-established questionnaire. This information was obtained through medical documentation and comprehensive interviews with the deceased person's family and friends, after receiving consent in the proforma prescribed by the Institutional Ethics Committee (IEC) from the deceased person's legal guardians. Subsequently, the gathered information was categorized according to different criteria in line with the objectives of this study, i.e., to study the patterns and probable reasons for dowry deaths in reproductive age groups. A standardized autopsy process (using I-shaped, Y-shaped, and modified Y-shaped incisions as per the demands of the case) was followed and, subsequently, the removal of organs was done using Virchow's technique, with findings being accurately documented.

A copy of the raw dataset was saved and never changed. Discussions along with scrutiny regarding data entry mistakes and bad data were done to facilitate fruitful analysis followed by encoding of specific raw data to enhance coherent analysis. Microsoft Excel was then employed in the development of a template aimed at inputting pertinent data, subsequently followed by examination using SPSS Statistics (IBM Corp., Armonk, NY) for Windows, and generation of frequency tables and proportions to examine the descriptive distribution of variables. This manuscript has been approved by IEC, RIMS, Ranchi, Jharkhand vide Memo no. 170/IEC, RIMS, Dated 27.03.2021. In the context of India, the need for consent is considered redundant when carrying out medicolegal autopsies due to their obligatory nature.

## Results

Among the fatalities associated with dowry, the deceased females were chiefly within the age spectrum of 16 to 26 years, with a mean age of 22.5 ± 4.6 years. Most females identified as Hindus, accounting for 10 cases, whereas two were Muslim. The victims exhibited an equal probability of originating from either joint or nuclear familial structures, though there was a discernible inclination of the victims to emerge from rural locales. All the deceased belonged to the middle socioeconomic strata (socioeconomic status categories II and III), with the majority of the victims being housewives. The spouses (males) chiefly engaged in entrepreneurial ventures (independently or familial) and agricultural activities. Neither the victims nor their spouses possessed any recognized professional credentials. A significant proportion of marriages were arranged, with dowry expectations frequently articulated before the nuptials, and in half of the cases, the duration of marriage spanned up to two years. Individuals lacking offspring represented a substantial majority (Table 1).

**Table 1:** Distribution of cases according to sociodemographic factors ^*^Modified B. G. Prasad classification for May 2022 [13]

Variable		N	%	Total	
				N	%
Age, years	15-20	05	41.67	12	100
	>20-25	05	41.67		
	>25-30	01	8.33		
	>30	01	8.33		
Religion	Hindu	10	83.33	12	100
	Muslim	02	16.67		
Family type	Nuclear	06	50	12	100
	Joint	06	50		
Residence	Rural	07	58.33	12	100
	Urban	05	41.67		
Socioeconomic status^*^	I	00	00	12	100
	II	09	75		
	III	03	25		
	IV	00	00		
	V	00	00		
Occupation of the deceased	Housewife	08	66.67	12	100
	Laborer	03	25		
	Farmer	01	8.33		
Occupation of the spouse	Farmer	04	33.33	12	100
	Own business	03	25		
	Family business	03	25		
	Service	02	16.67		
Education of the deceased	Primary school	03	25	12	100
	Middle school	04	33.33		
	Higher secondary	03	25		
	Intermediate	02	16.67		
	Graduate	00	00		
	Any professional degree	00	00		
Education of the spouse	Primary school	00	00	12	100
	Middle school	00	00		
	Higher secondary	05	41.67		
	Intermediate	03	25		
	Graduate	04	33.33		
	Any professional degree	00	00		
Type of marriage	Arranged	09	75	12	100
	Love	03	25		
Dowry demand before marriage	Yes	08	66.67	12	100
	No	04	33.33		
Duration of marriage, months	0-12	05	41.67	12	100
	>12-24	01	8.33		
	>24	06	50		
Number and gender of children	No children	06	50	12	100
	Only female child	03	25		
	Only male child	01	8.33		
	Both male and female child	02	16.67		

**Figure 1:**
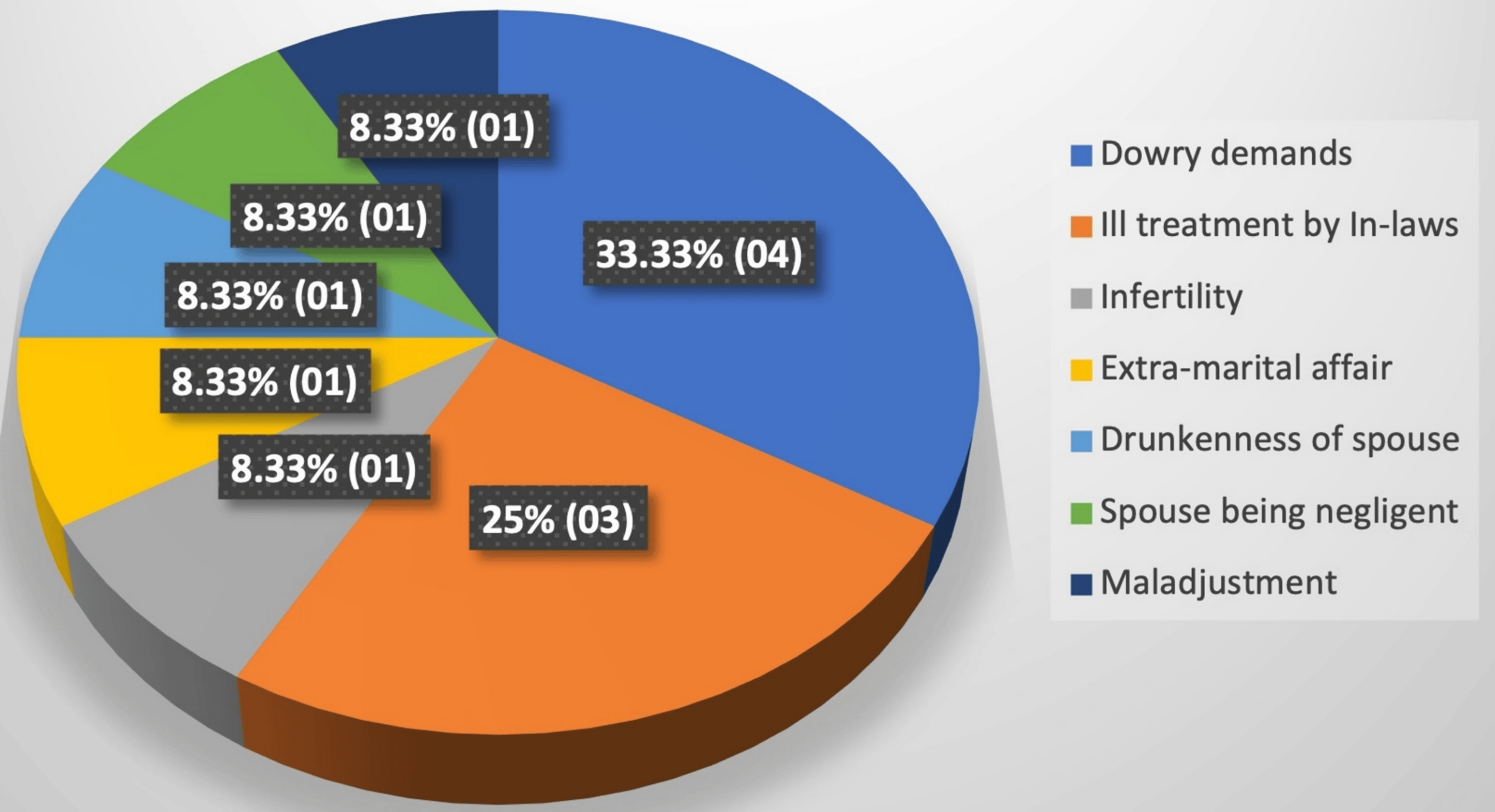
Distribution of cases according to the triggering factors behind suicidal and homicidal deaths

In several cases, the provocative factors were identified as financial expectations related to dowries and abusive treatment perpetrated by family members of the spouse (Figure 1).

**Figure 2:**
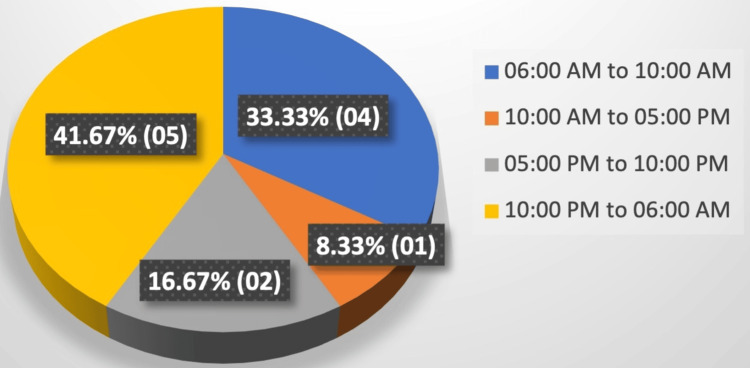
Distribution of cases according to the time of occurrence of instances leading to deaths

The majority of fatalities were recorded at night time, followed by the early morning timeframe (Figure 2).

The manner of death was homicidal in seven cases, suicidal in four cases, and remained inconclusive in one case. Thermal injuries emerged as the main cause of death in five scenarios, while stab wounds were the main reason in three scenarios. Two instances each were associated with firearm-related injuries and asphyxiation by hanging (Table 2).

**Table 2:** Distribution of cases according to the cause and manner of death

Cause of death	Manner of death							
	Homicidal		Suicidal		Inconclusive		Total	
	N	%	N	%	N	%	N	%
Burns	02	16.67	02	16.67	01	8.33	05	41.67
Hanging	00	00	02	16.67	00	00	02	16.67
Firearm	02	16.67	00	00	00	00	02	16.67
Stab	03	25	00	00	00	00	03	25
Total	07	58.33	04	33.33	01	8.33	12	100

The majority of the deaths occurred either within the household of the in-laws or at the residence of the partner (Table 3).

**Table 3:** Distribution of cases according to the location of incidence leading to dowry death and cause of death

Place of incidence	Cause of death								Total	
	Burns		Hanging		Firearm		Stabbing			
	N	%	N	%	N	%	N	%	N	%
House of In-laws (Sasural)	03	25	01	8.33	00	00	01	8.33	05	41.67
Spouse’s house	02	16.67	00	00	01	8.33	01	8.33	04	33.33
Own parent’s house (Mayaka)	00	00	01	8.33	00	00	00	00	01	8.33
Other places	00	00	00	00	01	8.33	01	8.33	02	16.67
Total	05	41.67	02	16.67	02	16.67	03	25	12	100

The parties presumed to be responsible for the act of either instigating the demise or coercing the individual into suicide and offenders in homicides in a considerable proportion of instances were the victim's relatives by marriage and their partner, with the latter frequently operating independently (Table 4).

**Table 4:** Distribution of cases according to the type of offenders and cause of death

Type of offenders/instigators	Cause of death								Total	
	Burns		Hanging		Firearm		Stabbing			
	N	%	N	%	N	%	N	%	N	%
Spouse	02	16.67	01	8.33	00	00	02	16.67	05	41.67
Spouse and in-laws	02	16.67	01	8.33	02	16.67	01	8.33	06	50
Other relatives of the spouse	01	8.33	00	00	00	00	00	00	01	8.33
Total	05	41.67	02	16.67	02	16.67	03	25	12	100

The majority of mortalities transpired during the treatment process within medical establishments, followed by fatalities occurring at the location of the criminal act, and during the transit to medical facilities (Table 5).

**Table 5:** Distribution of cases according to the place of death and cause of death

Place of death	Cause of death								Total	
	Burns		Hanging		Firearm		Stabbing			
	N	%	N	%	N	%	N	%	N	%
Death in the healthcare facility during treatment	03	25	01	8.33	00	00	01	8.33	05	41.67
Death on the spot	01	8.33	01	8.33	01	8.33	02	16.67	05	41.67
Death en route to the healthcare facility	01	8.33	00	00	01	8.33	00	00	02	16.67
Total	05	41.67	02	16.67	02	16.67	03	25	12	100

Sanitary pads or clothes were observed in situ among five victims, particularly in instances involving self-inflicted burns and hangings. None of the victims were pregnant at the time of their demise (Table 6).

**Table 6:** Distribution of cases according to the menstrual cycle and cause of death

Menstrual cycle	Cause of death								Total	
	Burns		Hanging		Firearm		Stabbing			
	N	%	N	%	N	%	N	%	N	%
Menstruating	02	16.67	02	16.67	00	00	01	8.33	05	41.67
Non-menstruating	03	25	00	00	02	16.67	02	16.67	07	58.33
Total	05	41.67	02	16.67	02	16.67	03	25	12	100

## Discussion

Dowry deaths, a severe indication of gender disparity, continue to pose a significant challenge in India and other South Asian nations, notwithstanding legislative bans such as the Dowry Prohibition Act of 1961 and sections 304B IPC (80 BNS) and 498A IPC (84 BNS) [14]. This occurrence is firmly entrenched in sociocultural customs in which dowry, defined as the exchange of money, assets, or presents from the bride's family to the groom's, is nearly universally observed even though it is illegal [15].

Economic development has exhibited a paradoxical impact on dowry-related fatalities; while certain regions have witnessed a decline in such occurrences alongside enhanced economic advancement, others have encountered a surge, indicating that established customs dictate conduct at lower economic strata, yet contemporary formal structures take precedence as progress is made [2]. The endurance of dowry-related fatalities is also ascribed to the intricate interaction of socioeconomic factors, encompassing the kinship network, the prevalence of extended families, women's engagement in the workforce, income disparity, and the effectiveness of the state's institutions [15].

The present study seeks to enhance the understanding of the sociodemographic determinants linked to dowry-related fatalities, in addition to factors concerning marriage, inciting elements, and other characteristics relating to the causes and circumstances surrounding such incidents within the context of the state of Jharkhand. In the majority of purported dowry-related fatalities, the deceased females were typically aged between 16 and 26 years. Numerous research studies have documented instances of dowry-related deaths within the age ranges of 18 to 26 years [16-19], 18 to 30 years [20-24], 21 to 30 years [25-27], 23 to 30 years [3], and 26 to 30 years [28], with some researchers reporting that a significant proportion of these tragic events occurred when the individuals were very young, specifically below 20 years of age [29], with possible child marriage implications.

The majority of the female individuals were affiliated with the Hindu faith, comprising 10 cases, while two cases were Muslim. The prevalence of Hindu victims in cases of dowry-related deaths has been highlighted in various studies [3,16-17,20-21,25,29], with some scholars revealing that all victims belonged to the Hindu community. Given the minority status of Muslims in India, the relatively lower incidence of dowry-related deaths among them could be attributed to their smaller representation in the total population [30]. Marriage is a contract and not a sacrament as per Islamic law, and the applicable laws related to marriages in Muslims, multiple marriages, and divorces also influence the causes behind deaths in marriages, other than dowry demands.

There was an equal chance of the victims coming from either joint or nuclear families; the majority of the victims hailed from rural areas. Other studies have reported majority of the victims hailed from joint families and rural areas [16-18,23], with some authors documenting that most victims came from rural areas and were part of nuclear families [22], while some have noted a prevalence of urban areas and joint families [28]. All of the deceased females belonged to the middle socioeconomic strata (socioeconomic status grade II and III), as observed in numerous other research works [3,17-19,25]; however, it does not align with some studies in which majority of the casualties were from the lower socioeconomic class [20,27,29].

The majority of the victims were housewives, a trend consistent with several previous research studies [17-19,20,22,24,27-29]. The partners were primarily involved in businesses (own or family) and agricultural practices. Some scholars have observed a notable proportion of partners being also involved in business, with a significant proportion being jobless [23], with some observing a large number of partners being unemployed in the majority of instances [19]. In numerous prior research endeavors, the victims were identified as either lacking literacy skills [22-23,28] or falling into the categories of non-matriculate or matriculate [17-19,20,24-25,27,29]. Similarly, in the current investigation, it was observed that none of the victims possessed any form of professional qualification, a pattern that extended to their respective partners as well.

Most of the marriages were arranged, with dowry demands often made before the marriage, in line with previous research [26]. Half of the instances involved a marriage duration of up to two years, as also observed by some other scholars in their study [29], where 27% of the deaths occurred within the second year of marriage while 23% of the victims died within the first year. Other studies have also indicated the occurrence of deaths within the first year [27], three years [16,19,22,28], and up to four years [18,20-21,23-24]. In a previous study, the majority of the individuals affected either had only female offspring or were childless [23]. This aligns with the current study, albeit with a distinction that individuals without children constituted the larger proportion. In previous studies, the triggering factors in several instances were dowry demands and mistreatment by In-laws [19,23], similar to the current study. Dowry (66.67% cases), followed by domestic violence (17.86% cases), emerged as the primary instigating factors in the research by some scholars [27], with some observing mistreatment by either the spouse, in-laws, or both as the principal instigating factor [18].

In previous research, the highest number of fatalities (72.86%) occurred during nighttime [18]. In this study, the most number of deaths were documented also during nighttime, followed by the morning. The majority of dowry deaths were determined to be homicidal (seven cases), while four cases were classified as suicidal, aligning with the research findings of some scholars [3]. Various studies have indicated that suicide was the most prevalent cause of death, followed by homicides [18-20,22,25,27], with some scholars demonstrating that suicide was the primary manner of death, followed by accidents and homicides [23-24,26]. Burns were identified as the cause of mortality in five cases, with stab injuries following closely (three cases), followed by firearm injuries and hanging (two cases each). Other studies have also highlighted burns as the most prevalent cause of fatality, followed by hanging and poisoning [16,19,20,25-26]. Some researchers have identified burn injuries followed by poisoning as the most prevalent causes of mortality [14,17,23,29]. Conversely, hanging was the most frequent method observed in the other studies, followed by incidents of burning and poisoning [18,21].

The location of occurrence in most fatalities was either the residence of the in-laws or the domicile of the spouse, mirroring the research of other scholars [25]. Similarly, some investigators have also found the primary site of occurrence being one's own residence or the residence of the in-laws [27]. In a significant proportion of cases involving dowry deaths, the individuals presumed to be accountable for the act of either causing the death or compelling the victim to commit suicide and offenders in homicides were found to be the victim's in-laws and spouse, with the latter often acting alone. This pattern is consistent with the findings of other researchers [18,25]. The majority of fatalities occurred during treatment within healthcare facilities, followed by the scene of the crime, and while being transported to healthcare facilities. It was similarly observed in earlier research that the majority of deaths occurred within healthcare facilities [25].

Sanitary pads or cloth were present in situ in five victims, particularly in cases of self-inflicted burns and hangings, similar to an earlier study [27]. Notably, none of the victims in the current study were pregnant at the time of their demise.

Limitations of the study

The study's generalizability is limited by the fact that it was conducted at a single tertiary healthcare institution. Also, there was a bias in the participant selection process, as it relied solely on cases of dowry-related fatalities that were autopsied at our institution. A comprehensive long-term study with a wider dataset with a multicentric approach, conducted in collaboration with disciplines in the realm of social sciences, is imperative to gain a deeper understanding of the complexities associated with this societal malice.

Recommendations

Dowry deaths remain a grave issue in India, necessitating multifaceted interventions to mitigate this social evil. The anti-dowry programs implemented by United Nations Women and Cooperative for Assistance and Relief Everywhere (CARE) International, focusing on advocacy, education, economy, and health, have shown promise in reducing gender discrimination and dowry-related deaths. However, the persistence of dowry deaths, particularly among young, unemployed women within the first few years of marriage, underscores the need for continuous legal and social reforms. The alarming statistics, such as the NCRB's report of an average of six dowry-related female suicides per day, highlight the inadequacy related to enforcing mechanisms of existing laws. To address this, it is crucial to enhance the legal framework by revising and strictly enforcing laws against dowry demands and related violence. Additionally, increasing public awareness and education about women's rights and the legal repercussions of dowry practices can empower women and deter potential offenders. 

The socioetiological factors, such as the high incidence of suicidal burns and the vulnerability of women in the early years of marriage, call for targeted interventions, including support systems for newly married women and stringent monitoring of dowry practices. Furthermore, the cultural and religious dimensions of dowry need to be addressed through community engagement and dialogue to shift societal norms. Overall, a combination of legal reforms, educational initiatives, and community-based approaches is essential to eradicate dowry deaths and protect women's rights in India.

## Conclusions

Our findings show that a significant number of individuals in suspected dowry-related deaths who underwent postmortem examinations fell within the 16-to-26-year age bracket and were Hindu by faith, with an equal likelihood of their coming from either joint or nuclear families, as well as an almost identical likelihood of originating from rural or urban settings. Predominantly, the marriages were arranged, and a dowry request of some kind was typically articulated before the marriage, with half of these marriages lasting no more than two years. The deceased predominantly did not have offspring and belonged to the middle socioeconomic stratum, with a majority of them being housewives and lacking any higher education. Conversely, their spouses, who were predominantly engaged in businesses and agricultural pursuits, also lacked professional qualifications.

The majority of dowry-related deaths exhibited homicidal characteristics, followed by cases of suicides, with burn injuries emerging as the most prevalent cause of fatality, followed by stab wounds, firearm wounds, and instances of hanging. A significant portion of these incidents occurred either at the residence of the in-laws or the husband's domicile, with the in-laws and the husband themselves being identified as the primary perpetrators in most scenarios, wherein they either directly caused the death or coerced the victim into taking her own life, typically triggered by demands for dowry and maltreatment by the in-laws. A notable proportion of fatalities transpired during medical treatment in healthcare institutions, followed by deaths at the scene of the crime, with most occurrences transpiring during the nighttime, followed by the morning hours. The findings underscore the pressing need for more concerted efforts by various stakeholders to eliminate this heinous menace from Indian society.
